# The Impact of Bariatric Surgery on Breast Cancer Recurrence: Case Series and Review of Literature

**DOI:** 10.1007/s11695-019-04099-6

**Published:** 2020-02

**Authors:** Shijia Zhang, Sayeed Ikramuddin, Heather C. Beckwith, Adam C. Sheka, Keith M. Wirth, Anne H. Blaes

**Affiliations:** 1 Division of Hematology, Oncology and Transplantation, Department of Medicine, University of Minnesota, 420 Delaware Street, SE, MMC 480, Minneapolis, MN 55455, USA; 2 Department of Surgery, University of Minnesota, Minneapolis, MN, USA

**Keywords:** Bariatric surgery, Weight loss, Breast cancer, Recurrence

## Abstract

**Background:**

Excess body weight has been associated with worsening breast cancer survival. While bariatric surgery has been associated with less incident of breast cancer, the role that bariatric surgery plays after breast cancer diagnosis in terms of both feasibility and in preventing breast cancer recurrence is unclear.

**Methods:**

We report the outcomes of 13 individuals who underwent bariatric surgery after definitive breast cancer treatment at a single institution.

**Results:**

Thirteen females diagnosed with breast cancer (69.2% stage I, 23.1% stage II) at a median age of 42 years received bariatric surgery between 2001 and 2017. The median age of bariatric surgery was 52 years. Of the 13 patients, 46.2% underwent laparoscopic Roux-en-Y gastric bypass and 38.5% laparoscopic sleeve gastrectomy. The median time from breast cancer treatment to bariatric surgery was 3 years. The procedures were well tolerated. One female developed an abdominal wall hematoma. The average weight loss after 1 year and 2 years was 28.1% and 28.2%, respectively. There was a single breast cancer recurrence with a median follow-up of 11.7 years after breast cancer diagnosis and 5.3 years after bariatric surgery.

**Conclusions:**

Bariatric surgery after breast cancer treatment is feasible and well tolerated. Prospective trials evaluating bariatric surgery in obese breast cancer survivors should be considered.

## Introduction

It is estimated that overweight and obesity could account for 14% of all deaths from cancer in males and 20% of those in females in the USA [1]. For those with a body mass index (BMI) between 27.5–29.9 kg/m^2^, the risk of cancer increases by 12%, while those with a BMI over 40 kg/m^2^ have a 70% increased risk of cancer compared to those with a normal BMI [2]. On the contrary, dramatic weight loss from bariatric surgery is associated with reduced cancer mortality by approximately 40% [3]. This suggests that promoting healthy weight change in adults can have important health benefits and outcomes from a cancer perspective.

Our interest is to look at the impact of weight loss on breast cancer survivors. A recent meta-analysis of 82 studies that included 213,075 women with breast cancer demonstrated that for each 5 kg/m^2^ increment in BMI, there was a 14 to 29% increased risk of breast cancer–specific mortality and an 8 to 17% increased risk of overall mortality [4]. Multiple trials have been initiated to look at the impact of lifestyle intervention and dietary modification to produce weight loss in patients with a history of breast cancer. The results of these trials have been disappointing due to poor compliance with the proposed intervention producing only modest weight loss and questionable results in terms of local recurrence improvement. Bariatric surgery has been shown to be the most effective tool to achieve and maintain long-term weight loss. In a large multisite cohort study, the risk of postmenopausal breast cancer was significantly lower (hazard ratio [HR] 0.58, 95% confidence interval [CI] 0.44–0.77, *P* < 0.001) among patients who had undergone bariatric surgery compared with matched nonsurgical controls [5]. While bariatric surgery can improve obesity-related health problems, such as type 2 diabetes, hypertension, and sleep apnea, the impact on recurrence of breast cancer is unclear. The aim of this study was to examine the safety of bariatric surgery after breast cancer treatment on breast cancer recurrence. Here, we report the outcomes of 13 patients, who had bariatric surgery after definitive breast cancer therapy.

## Methods

Following approval from the Institutional Review Board of our institution, a computerized search from the electronic medical records of the University of Minnesota and Fairview Health Systems was performed for patients who have had a diagnosis of breast cancer and underwent bariatric surgery from 2001 to 2017. Medical records of patients who had definitive breast cancer treatment prior to bariatric surgery were reviewed for data collection. Descriptive statistics were used to describe the features of the data.

## Case Series

Patients included in this analysis had to meet the following two criteria: (1) had bariatric surgery (Roux-en-Y gastric bypass, sleeve gastrectomy, adjustable banding, or duodenal switch) between 2001 and 2017, and (2) had definitive treatment of breast cancer prior to bariatric surgery (Table 1). A total of 13 patients met the study criteria. All were female. The median age of initial breast cancer diagnosis was 42 (range 30–57) years. At the time of breast cancer diagnosis, 2 (15.4%) patients had BMI in the range of 30–34.9 kg/m^2^, 4 (30.8%) in the range of 35–39.9 kg/m^2^, 4 (30.8%) in range of 40 kg/m^2^ and above, and 3 (23.1%) with unknown BMI. At least 10 (76.9%) of these patients were obese (BMI ≥ 30 kg/m^2^) when they were diagnosed with breast cancer. Nine (69.2%) patients had stage I breast cancer and 3 (23.1%) had stage II disease. The breast cancer staging of 1 (7.7%) patient was unknown. One (7.7%) patient had a tumor that was hormone receptor (HR)–positive and human epidermal growth factor receptor 2 (HER2)–positive, 9 (69.2%) HR-positive and HER2-negative, 2 (15.4%) triple negative, and 1 (7.7%) with unknown HR and HER2 status. All patients had surgery for breast cancer 6 (46.2%) patients underwent lumpectomy, 1 (7.7%) unilateral mastectomy, and 6 (46.2%) bilateral mastectomies. Six (46.2%) patients had radiation therapy, and 9 (69.2%) patients received adjuvant chemotherapy. Ten (76.9%) patients were treated with adjuvant endocrine therapy.

**Fig. 1:**
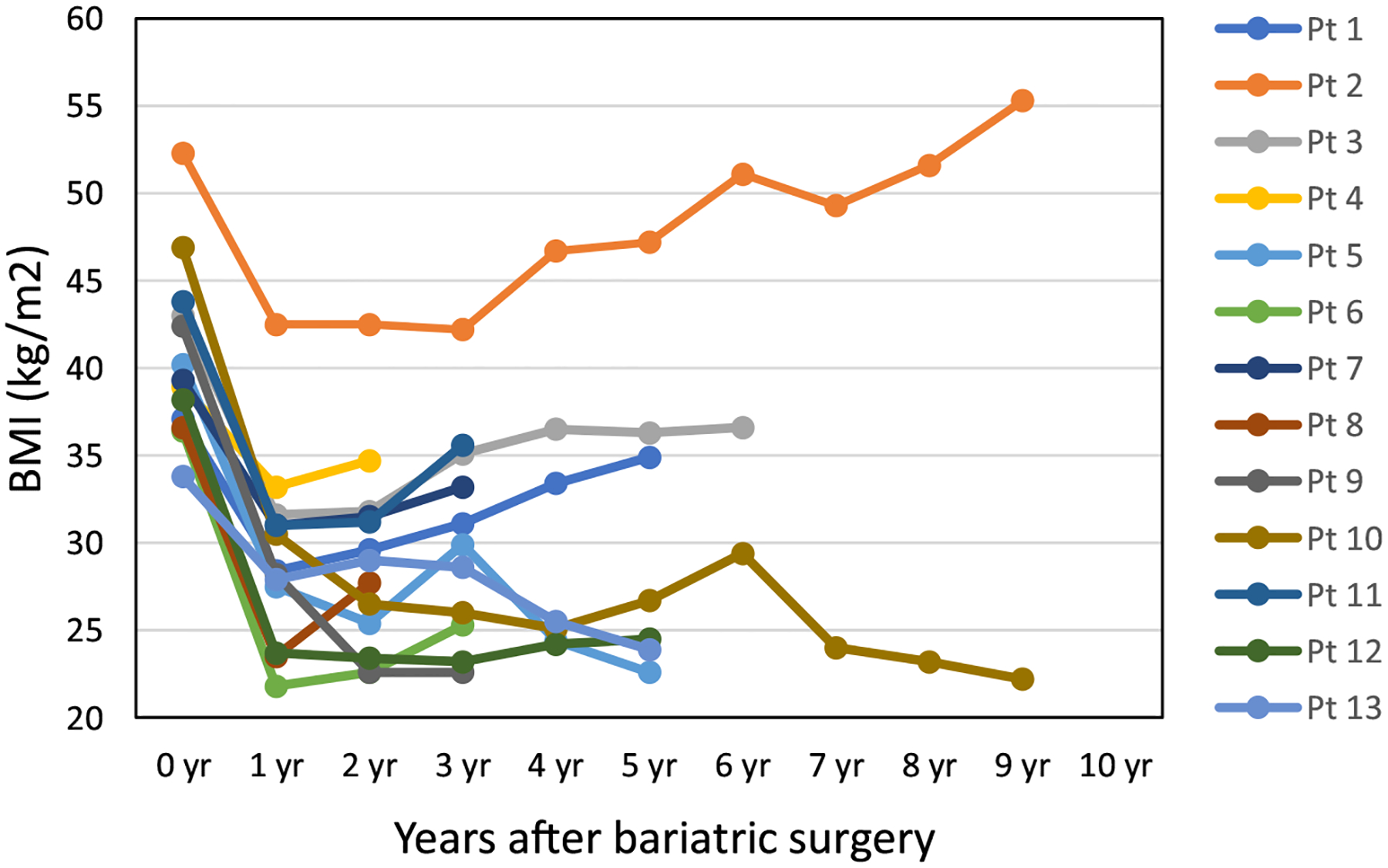
Body mass index (BMI) at the time of bariatric surgery and yearly after bariatric surgery

The median age at bariatric surgery was 52 (range 33–63) years. The median time from breast cancer diagnosis to bariatric surgery was 3 (range 1.1–14.2) years. At the time of bariatric surgery, 1 (7.7%) patient had BMI in the range of 30–34.9 kg/m^2^, 6 (46.2%) in the range of 35–39.9 kg/m^2^, and 6 (46.2%) in the range of 40 kg/m^2^ and above. For those with available BMI at breast cancer diagnosis, 7 out of 10 (70%) gained weight between the time of breast cancer diagnosis and the time of bariatric surgery. Six (46.2%) patients underwent laparoscopic Roux-en-Y gastric bypass, 5 (38.5%) laparoscopic sleeve gastrectomy, 1 (7.7%) laparoscopic adjustable gastric band, and 1 (7.7%) laparoscopic duodenal switch. These procedures were generally well tolerated by the patients. In the postoperative period, only 1 (7.7%) patient developed abdominal wall hematoma in the camera trocar site resulting in a large hemoglobin drop that required red blood cell transfusions. For most patients, the maximal weight loss occurred within 2 years of post-bariatric surgery (Table 2). The average weight loss after 1 year and 2 years was 28.1% (range 17.5–40.1%) and 28.2% (range 10.8–46.7%), respectively. Furthermore, most patients maintained durable weight loss (Fig. 1).

There was a single breast cancer recurrence with a median follow-up of 11.7 (range 3.9–20.6) years after breast cancer diagnosis and 5.3 (range 2.0–9.8) years after bariatric surgery. This patient was diagnosed with stage IIA (T2N0M0), estrogen receptor (ER)-positive, progesterone receptor (PR)-positive, and HER-2-negative breast cancer at age 35. She was found to be a carrier of BRCA2 mutation. She underwent left mastectomy followed by chemotherapy (cyclophosphamide, methotrexate, 5-fluorouracil). She was started on tamoxifen and then switched to exemestane after she had total abdominal hysterectomy and bilateral salpingo-oophorectomy at age 40. She underwent laparoscopic Roux-en-Y gastric bypass at age 43 when her BMI was 40.2 kg/m^2^. About 4 weeks after her bariatric surgery, she was found to have local recurrence of breast cancer and then metastatic disease. She has received multiple lines of therapy and has been doing well.

## Discussion

As the worldwide obesity epidemic spreads, we are facing more and more challenges from diabetes, cardiovascular diseases, cancers, and other health conditions related to obesity. Not only is the likelihood of development of breast cancer linked to obesity; but equally as concerning is the higher rate of reoccurrence following definitive therapy for breast cancer. A meta-analysis of 43 studies showed that women who were obese at breast cancer diagnosis had an approximately 33% higher risk of mortality compared with normal-weight women [6]. Attempts have been made to conduct prospective interventional studies to look at the impact of weight loss on overweight breast cancer survivors. The Lifestyle Intervention in Adjuvant Treatment of Early Breast Cancer (LISA) trial randomized overweight breast cancer survivors receiving adjuvant letrozole to mail-based delivery of general health information ± telephone-based lifestyle interventions over a period of 24 months. The primary endpoint was disease-free survival. Unfortunately, the enrollment was discontinued early at 338 of 2,150 planned participants because of loss of funding. The average weight loss in the lifestyle interventions arm was 5.5 vs 0.7% in the control arm after 1 year, and 3.6% vs 0.4% after 2 years. The impact of this modest weight loss on breast cancer recurrence and mortality remains unknown [7].

Bariatric surgery is the most robust and durable therapy for obesity and its related complications. As shown in the LISA study, the amount of weight loss mediated by lifestyle intervention is usually modest (3.6% after 2 years). On the contrary, in our cohort of breast cancer survivors, bariatric surgery induced significantly greater and durable weight loss (28.2% after 2 years) in most patients. In the Diabetes Surgery Study Randomized Clinical Trial, patients in the Roux-en-Y gastric bypass group (*N* = 60) lost 26.1% vs 7.9% of their initial body weight at 1 year compared with the lifestyle-medical management group (*N* = 60). The weight loss effect persisted at 5 years: 21.8% in the Roux-en-Y gastric bypass group vs 9.6% in the lifestyle-medical management group in an intent-to-treat analysis (15% of the lifestyle intervention patients actually ended up getting bariatric surgery) [8].

Analysis of the International Breast Cancer Study Group clinical trials I–V with 4105 eligible participants showed that the breast cancer-free rate was around 43% for patients with ER-positive disease and 46% for those with ER-negative disease 12 years from random assignment (estimated from the breast cancer-free interval curves), indicating high breast cancer recurrence rate [9]. In our cohort, the disease recurrence rate was 7.7% (1 out of 13 patients) with a median follow-up of 11.7 years. It is noteworthy that this patient was found to have breast cancer recurrence only 4 weeks after her Roux-en-Y gastric bypass surgery. Therefore, it was likely too soon to evaluate the impact of bariatric surgery. Also, she carries a BRCA2 mutation and only had unilateral mastectomy, making her at high risk for recurrent or new breast cancer.

Bariatric surgery appears to be relatively safe in patients who had definitive breast cancer treatment—only 1 (7.7%) patient in our cohort had bleeding in the abdominal wall in the postoperative period. Given the small sample size, it would not be feasible to compare the postoperative complication rate with other large studies. In a study of 268,898 metabolic and bariatric surgeries performed between 2007 and 2010, the 30-day serious complication rate was 1.25% for gastric bypass, 0.96% for sleeve gastrectomy, and 0.25% for gastric banding. The authors did not define “serious complication” in their abstract, and it is unclear if abdominal hematoma would qualify for “serious complication” [10].

This study is limited by small sample size, retrospective analysis, and no patients with stage III breast cancer at diagnosis. Although it appears relatively safe to have bariatric surgery for breast cancer survivors and most of these patients seem to have a good outcome from a breast cancer perspective based on our study, larger studies are required to confirm these results. Recently, we conducted a retrospective cohort study of breast cancer patients undergoing bariatric surgery 2004–2017 using de-identified data from a large U.S. commercial insurance database (OptumLabs®Data Warehouse). The relative risk of breast cancer events for patients who underwent bariatric surgery was 45% lower than the non-surgical group [11]. With these results, proposing bariatric surgery for obese breast cancer survivors in an effort not only to reduce comorbidities such as diabetes and hypertension but also to help prevent breast cancer recurrence should be considered [12].

## Conclusion

In obese breast cancer survivors, weight management strategies should be utilized to prevent recurrence. Our results suggest that bariatric surgery is well-tolerated in breast cancer patients who have undergone definitive treatment for their malignancy. As bariatric surgery is significantly more effective in producing long-term weight loss than lifestyle management, physicians should discuss this option with obese breast cancer patients and refer appropriate patients to a bariatric surgeon as part of ongoing preventative therapy. A randomized control trial of bariatric surgery versus best medical management in obese breast cancer survivors should be considered.

## Figures and Tables

**Table 1.** Characteristics of patients in our cohort

No.	Age at BC dx	BMI at BC dx	Stage	ER +	PR +	HER2 +	BC surety	CT	CT regimen	RT	ET	Age at BS	Yrs from BC dx to BS	BMI at BS	Type of BS	Post-op complications from BS	% wt loss in 1 yr	% wt loss in 2 yrs	BC recurrence
1	50	32.6	IA	Y	N	N	Right lumpectomy	N	–	Y	Y	56	6.0	37.1	Lap sleeve gastrectomy	None	23.5	20.2	N
2	30	52.6	HA	N	N	N	Bilateral mastectomies	Y	Doxorubicin + cyclophosphamide then paclitaxel	N	N	33	3.0	52.3	Lap adjustable gastric band	None	18.7	18.7	N
3	47	37.8	IA	Y	Y	N	Left lumpectomy	Y	Doxorubicin + cyclophosphamide	Y	Y	52	5.5	43	Lap sleeve gastrectomy	None	26.5	26.0	N
4	57	39.4	IA	Y	Y	N	Bilateral mastectomies	N	–	N	Y	59	2.7	38.9	Lap Roux-en-Y gastric bypass	Abdominal wall hematoma	14.7	10.8	N
5	35	NA	HA	Y	Y	N	Left mastectomy	Y	Cyclophosphamide + methotrexate +5-fluorouracil	N	Y	43	7.5	40.2	Lap Roux-en-Y gastric bypass	None	31.6	36.8	Y
6	50	31.0	IA	Y	Y	N	Bilateral mastectomies	Y	Cyclophosphamide + paclitaxel	N	Y	52	2.3	36.4	Lap sleeve gastrectomy	None	40.1	37.9	N
7	36	36.9	IA	Y	Y	Y	Right lumpectomy	Y	Doxorubicin + cyclophosphamide	Y	Y	45	9.2	39.3	Lap sleeve gastrectomy	None	21.1	19.8	N
8	42	35.0	HA	Y	Y	N	Left lumpectomy	Y	Paclitaxel then doxorubicin + cyclophosphamide	Y	Y	43	1.3	36.6	Lap sleeve gastrectomy	None	35.8	24.3	N
9	42	44.0	IA	Y	Y	N	Bilateral mastectomies	N	–	N	Y	43	1.1	42.4	Lap Roux-en-Y gastric bypass	None	33.5	46.7	N
10	41	44.3	IA	Y	Y	N	Bilateral mastectomies	Y	Cyclophosphamide + methotrexate +5-fluorouracil	N	Y	44	2.2	46.9	Lap Roux-en-Y gastric bypass	None	35.0	43.5	N
11	53	43.7	IA	Y	Y	N	Right lumpectomy	N	–	Y	Y	56	2.4	43.8	Lap Roux-en-Y gastric bypass	None	29.2	28.8	N
12	42	NA	NA	NA	NA	NA	Left lumpectomy	Y	NA	Y	NA	55	13.3	38.2	Lap duodenal switch	None	38.0	38.7	N
13	49	NA	IA	N	N	N	Bilateral mastectomies	Y	NA	N	N	63	14.2	33.8	Lap Roux-en-Y gastric bypass	None	17.5	14.2	N

*BC*, breast cancer; *BMI*, body mass index; *BS*, bariatric surgery; *CT*, chemotherapy, *dx*, diagnosis; *ET*, endocrine therapy, *lap*, laparoscopic; *NA*, not available; *RT*, radiation therapy, *wt*, weight; *yr*, year

**Table 2.** Descriptive statistics of our cohort

Characteristic	Overall (*N* = 13)
Age, median (range)	
At breast cancer diagnosis	42 (30–57)
At bariatric surgery	52 (33–63)
Years from BC dx to BS, median (range)	3.0 (1.1–14.2)
BMI at breast cancer diagnosis, *N* (%)	
30–34.9	2 (15.4)
35–39.9	4 (30.8)
40 and above	4 (30.8)
Missing/NA	3 (23.1)
BMI at bariatric surgery, *N* (%)	
30–34.9	1 (7.7)
35–39.9	6 (46.2)
40 and above	6 (46.2)
Type of bariatric surgery, *N* (%)	
Laparoscopic Roux-en-Y gastric bypass	6 (46.2)
Laparoscopic sleeve gastrectomy	5 (38.5)
Laparoscopic adjustable gastric band	1 (7.7)
Laparoscopic duodenal switch	1 (7.7)
Post-op complication, *N* (%)	
None	12 (92.3)
Abdominal wall hematoma	1 (7.7)
Breast cancer stage at diagnosis, *N* (%)	
I	9 (69.2)
II	3 (23.1)
Missing/NA	1 (7.7)
Hormonal receptor (HR)/HER-2 status, *N* (%)	
HR (+), HER-2 (+)	1 (7.7)
HR (+), HER-2 (−)	9 (69.2)
HR (−), HER-2 (−)	2 (15.4)
Missing/NA	1 (7.7)
Breast cancer surgery type, *N* (%)	
Lumpectomy	6 (46.2)
Unilateral mastectomy	1 (7.7)
Bilateral mastectomies	6 (46.2)
Treated with adjuvant chemotherapy, *N* (%)	
Yes	9 (69.2)
No	4 (30.8)
Treated with radiation therapy, *N* (%)	
Yes	6 (46.2)
No	7 (53.8)
Treated with endocrine therapy, *N* (%)	
Yes	10 (76.9)
No	2 (15.4)
Missing/NA	1 (7.7)
Recurrence of breast cancer, *N* (%)	
Yes	1 (7.7)
No	12 (92.3)

*BC*, breast cancer; *BMI*, body mass index; *BS*, bariatric surgery; *dx*, diagnosis; *NA*, not available
